# Incorporation of transition to transversion ratio and nonsense mutations, improves the estimation of the number of synonymous and non-synonymous sites in codons

**DOI:** 10.1093/dnares/dsac023

**Published:** 2022-08-03

**Authors:** Ruksana Aziz, Piyali Sen, Pratyush Kumar Beura, Saurav Das, Debapriya Tula, Madhusmita Dash, Nima Dondu Namsa, Ramesh Chandra Deka, Edward J Feil, Siddhartha Sankar Satapathy, Suvendra Kumar Ray

**Affiliations:** Department of Molecular Biology and Biotechnology, Tezpur University, Tezpur, 784028 Assam, India; Department of Computer Science and Engineering, Tezpur University, Tezpur, 784028 Assam, India; Department of Molecular Biology and Biotechnology, Tezpur University, Tezpur, 784028 Assam, India; Department of Molecular Biology and Biotechnology, Tezpur University, Tezpur, 784028 Assam, India; TCS Innovation, Tata Consultancy Services, Hyderabad, 500081 Telangana, India; Department of Electronics and Communication Engineering, NIT, Papum Pare, 791113 Arunachal Pradesh, India; Department of Molecular Biology and Biotechnology, Tezpur University, Tezpur, 784028 Assam, India; Center for Multidisciplinary Research, Tezpur University, Tezpur, 784028 Assam, India; Center for Multidisciplinary Research, Tezpur University, Tezpur, 784028 Assam, India; Department of Chemical Sciences, Tezpur University, Tezpur, 784028 Assam, India; Department of Biology and Biochemistry, The Milner Centre for Evolution, University of Bath, Bath BA2 7AY, UK; Department of Computer Science and Engineering, Tezpur University, Tezpur, 784028 Assam, India; Center for Multidisciplinary Research, Tezpur University, Tezpur, 784028 Assam, India; Department of Molecular Biology and Biotechnology, Tezpur University, Tezpur, 784028 Assam, India; Center for Multidisciplinary Research, Tezpur University, Tezpur, 784028 Assam, India

**Keywords:** dN/dS, synonymous/non-synonymous sites, pretermination codon, transition, transversion

## Abstract

A common approach to estimate the strength and direction of selection acting on protein coding sequences is to calculate the dN/dS ratio. The method to calculate dN/dS has been widely used by many researchers and many critical reviews have been made on its application after the proposition by Nei and Gojobori in 1986. However, the method is still evolving considering the non-uniform substitution rates and pretermination codons. In our study of SNPs in 586 genes across 156 *Escherichia coli* strains, synonymous polymorphism in 2-fold degenerate codons were higher in comparison to that in 4-fold degenerate codons, which could be attributed to the difference between transition (Ti) and transversion (Tv) substitution rates where the average rate of a transition is four times more than that of a transversion in general. We considered both the Ti/Tv ratio, and nonsense mutation in pretermination codons, to improve estimates of synonymous (S) and non-synonymous (NS) sites. The accuracy of estimating dN/dS has been improved by considering the Ti/Tv ratio and nonsense substitutions in pretermination codons. We showed that applying the modified approach based on Ti/Tv ratio and pretermination codons results in higher values of dN/dS in 29 common genes of equal reading-frames between *E. coli* and *Salmonella enterica*. This study emphasizes the robustness of amino acid composition with varying codon degeneracy, as well as the pretermination codons when calculating dN/dS values.

## 1. Introduction

A common approach to estimate the strength and direction of selection acting on protein coding sequences is to calculate the dN/dS ratio where dN is defined as the number of non-synonymous (NS) changes per non-synonymous site, whereas dS is defined by the number of synonymous changes (S) per synonymous sites in the gene sequences.^1–3^ The number of synonymous sites (or non-synonymous sites) in a codon is simply calculated as the fraction of all three possible changes that are synonymous (or non-synonymous) at a given codon position and summing over all three positions.^4^^,^^5^ Every position/site of one codon can undergo three possible changes resulting nine possible site changes for the given codon. Any change at a given site can be either S or NS. If all the changes at a particular site result in S change, the site is a synonymous site. For instance, at the second site of any codon in the genetic code table will result a NS change, hence the site will be termed as NS site and the value of NS site is one. But, when one out of three changes at first or third site is S change and other two changes results in NS change the site value becomes one-third for S site, i.e. 0.33 S and two-third for NS site, i.e. 0.67 NS (Table 1). The method to calculate dN/dS has been widely used by many researchers and many critical reviews have been made on its application after the proposition by Gojobori and Nei.^3^^,^^6–8^ In their model, the dN and dS were estimated by applying Jukes and Cantor’s formula,^9^^,^^10^ where the rate of different substitutions is considered equal. But, the non-uniform substitution rates between transition and transversion proposed by Kimura,^11^ were not considered to estimate the dN/dS ratio.^12^ In addition, Hasegawa *et al*.^13^ had postulated unequal rate among the different substitutions that further suggest the importance of transition and transversion rate difference for the estimation of dN/dS. Eventually, using computer simulation detail analysis of estimating S and NS sites in codons proposed by different researchers was studied.^14^

**Table 1:** S and NS sites of codons in the genetic code table by the original method

Old method	U		C		A		G		
	S	NS	S	NS	S	NS	S	NS	
U	0.333	2.667	1.000	2.000	0.333	2.667	0.333	2.667	U
	0.333	2.667	1.000	2.000	0.333	2.667	0.333	2.667	C
	0.667	2.333	1.000	2.000	N/A	N/A	N/A	N/A	A
	0.667	2.333	1.000	2.000	N/A	N/A	0.000	3.000	G
C	1.000	2.000	1.000	2.000	0.333	2.667	1.000	2.000	U
	1.000	2.000	1.000	2.000	0.333	2.667	1.000	2.000	C
	1.333	1.667	1.000	2.000	0.333	2.667	1.333	1.667	A
	1.333	1.667	1.000	2.000	0.333	2.667	1.333	1.667	G
A	0.667	2.333	1.000	2.000	0.333	2.667	0.333	2.667	U
	0.667	2.333	1.000	2.000	0.333	2.667	0.333	2.667	C
	0.667	2.333	1.000	2.000	0.333	2.667	0.667	2.333	A
	0.000	3.000	1.000	2.000	0.333	2.667	0.667	2.333	G
G	1.000	2.000	1.000	2.000	0.333	2.667	1.000	2.000	U
	1.000	2.000	1.000	2.000	0.333	2.667	1.000	2.000	C
	1.000	2.000	1.000	2.000	0.333	2.667	1.000	2.000	A
	1.000	2.000	1.000	2.000	0.333	2.667	1.000	2.000	G

Out of 12 substitution mutations, 8 are transversions (Tv) and 4 are transitions (Ti).^15^^,^^16^ However, because Ti mutations occur between bases with the same ring structure (purine to purine or pyrimidine to pyrimidine), these mutations tend to be ∼2-fold more common than transversions.^17–19^ For instance, in *Escherichiacoli*, a Ti substitution is in average four times more frequent than a Tv substitution.^19^ Synonymous changes in 2-fold degenerate (TFD) codons are always linked by Ti; hence, mutations are anticipated to be accumulated faster in these codons than in 4-fold degenerate (FFD) codons, although synonymous changes can arise by either transitions or transversions. Apart from the impact of Ti/Tv ratio on S as well as NS changes, the nonsense mutation will have impact on NS in a codon. In the genetic code table, among the total 183 single substitutions in 61 sense codons, 23 substitutions in 18 different pretermination codons result in nonsense codons.^15^ The five codons in which two out of nine single substitutions resulting nonsense codons are UUA, UCA, UGG, UAU, and UAC. Unlike other missense mutations which alters the amino acid sequence in the encoded polypeptide at the site only, nonsense mutation not only influences at the site, but also it apparently converts all the downstream codons in the frame as nonsense codons. Therefore, nonsense mutation should be treated separately than other missense mutation while calculating NS site in codons.

The contribution of the Kimura’s two parameters along with the impact of pretermination codon in our modified method were analysed and the findings were compared with Nei–Gojobori method. Yang *et al*. also estimated dN/dS in genes using maximum likelihood approach, which accounts the substitutions rates in detail. However, our modified method not only accounts Ti/Tv ratio but also considers the nonsense substitutions to estimate non-synonymous sites due to pretermination codons in a gene sequence, which was not considered in the previous methods. Therefore, we believe that our modified method is easy to understand, straightforward, biologically relevant, and mathematically simple.

In this study, we have analysed single nucleotide polymorphisms (SNPs) in 586 genes across 156 genomes of *E. coli* strains. We have demonstrated that SNPs accumulate faster in TFD codons than FFD codons. This has been further confirmed by 6-fold degenerate codons, where the split box synonymous codons accumulate SNPs faster than the family box (FFD) codons. The effect of Ti and Tv rate difference on SNPs at synonymous sites suggest that the effective number of synonymous sites should be higher in TFD as these are linked by transitions. In other words, synonymous changes in FFD codons should on average have a lower rate than those in TFD codons. Therefore, calculation of S and NS sites in codons should account the Ti and Tv rate difference. This will be important further to estimate the dN/dS values. Apart from the Ti/Tv ratio, the difference between pretermination codon from the other codons is needed to be accounted while calculating the NS site of the codons. In this study, we have calculated S and NS sites in codons accounting Ti/Tv and nonsense substitutions in pretermination codons. Further, we have compared dN/dS calculated by our approach with the parameters used in MEGA-X.

## 2. Materials and methods

### 2.1. Expected synonymous and non-synonymous polymorphism in *E. coli* genes

We have carried out our investigation for SNPs based on computational analysis of 586 genes across 156 strains of *E. coli*.^20^ Although *E.coli* genome has ∼4,500 genes but within the genome dataset of 156 strains, we excluded genes showing length variation in sequence alignment or having any ambiguous bases. We classified all SNPs at first, second, and third codon positions to categorize them into synonymous transition, synonymous transversions, non-synonymous transition, and non-synonymous transversion. We used the observed numbers of synonymous and non-synonymous SNPs to calculate the expected synonymous (S_e_) and expected non-synonymous (NS_e_) changes for each codon. For examples, out of total 9 single substitutions in UUU codon, 1/9 is S and 8/9 is NS. Likewise, for individual codons in the genetic code table S and NS can be calculated out of total nine single substitutions. After finding the observed synonymous (S_o_) and non-synonymous (NS_o_) changes for each individual codon, S_e_ and NS_e_ are calculated by multiplying S_o_ (or NS_o_) with the S (or NS) of the corresponding codon. For example, if summation of S_o_ and NS_o_ for UUU is x and y, then 1/9 (x + y) is S_e_ change for UUU and 8/9 (x + y) is NS_e_ change for UUU. Using the same approach, S_e_ and NS_e_ for each codon were calculated. Comparison across the 59 codons have been performed by their S_o_/S_e_ values termed as fS (Supplementary Table S1). Calculation of synonymous site (S) and non-synonymous site (NS) in codons accounting Ti/Tv ratio, and pretermination codons. 

#### 2.1.1. Existing method


$$S=\frac{S_{\mathrm{ti}}+S_{\mathrm{tv}}}{3}$$

$$NS=\frac{N_{\mathrm{ti}}+N_{\mathrm{tv}}}{3}$$

#### 2.1.2. Modified method in this study


$$S=\frac{\kappa \times S_{\mathrm{ti}}+S_{\mathrm{tv}}}{\kappa +2}$$

$$NS=\frac{{\kappa \;\times N}_{\mathrm{ti}}+N_{\mathrm{tv}}}{\kappa +2}$$

$${NS}^{'}=\frac{{\kappa \;\times (N}_{\mathrm{ti}}-N_{\mathrm{ti}}^{'})+(N_{\mathrm{tv}}-N_{\mathrm{tv}}^{'})}{x+2}$$

where *S*, synonymous site of a codon; *NS*, non-synonymous site of a codon; κ, number of times a Ti is more frequent than a Tv; $S_{\mathrm{ti}},$ Synonymous transition due to single-site substitution in a codon; $S_{\mathrm{tv}}$, Synonymous transversion due to single-site substitution in a codon; $N_{\mathrm{ti}}$, non-synonymous transition due to single-site substitution in a codon; $N_{\mathrm{tv}}$, non-synonymous transversion due to single-site substitution in a codon; $N_{\mathrm{ti}}^{'}$, number of $N_{\mathrm{ti}}$ resulting to stop codon in a codon; $N_{\mathrm{tv}}^{'}$, number of $N_{\mathrm{tv}}$ resulting to stop codon in a codon.

The S/NS site calculated using the earlier method and the modified method proposed in this study for every codon are presented in Tables 1 and 2. For instance, the S site for CUG/CUA and CGA/CGG codons is considered 1.333 in earlier methods. Whereas in our modified method, the S site for CUA/CUG codons is calculated to be 1.667, and that for the CGA/CGG codons is 1.166. It is known that synonymous substitution in CUA/CUG codon is the highest among all the codons in the genetic code table because it involves two transition and two transversion substitutions, while synonymous substitution in case of CGA/CGG codon involves one transition and three transversion substitution.^21^ Further, the S site value of AUA codon in the earlier method is 0.667, whereas the S site value of the AUA codon in the modified method is 0.333, which is the least among the codons that can undergo synonymous substitutions in the genetic code table (Tables 1 and 2).

**Table 2:** S and NS sites of codons in the genetic code table by accounting a transition being four time more frequent than a transversion and the nonsense substitutions in the pretermination codons

New method	U		C		A		G		
	S	NS	S	NS	S	NS	S	NS	
U	0.667	2.333	1.000	2.000	0.667	2.000	0.667	2.167	U
	0.667	2.333	1.000	2.000	0.667	2.000	0.667	2.167	C
	1.333	1.333	1.000	1.667	N/A	N/A	N/A	N/A	A
	1.333	1.500	1.000	1.833	N/A	N/A	0.000	1.667	G
C	1.000	2.000	1.000	2.000	0.667	2.333	1.000	2.000	U
	1.000	2.000	1.000	2.000	0.667	2.333	1.000	2.000	C
	1.667	1.333	1.000	2.000	0.667	1.667	1.167	1.167	A
	1.667	1.333	1.000	2.000	0.667	1.667	1.167	1.833	G
A	0.833	2.167	1.000	2.000	0.667	2.333	0.667	2.333	U
	0.833	2.167	1.000	2.000	0.667	2.333	0.667	2.333	C
	0.333	2.667	1.000	2.000	0.667	2.167	0.833	2.000	A
	0.000	3.000	1.000	2.000	0.667	2.167	0.833	2.167	G
G	1.000	2.000	1.000	2.000	0.667	2.333	1.000	2.000	U
	1.000	2.000	1.000	2.000	0.667	2.333	1.000	2.000	C
	1.000	2.000	1.000	2.000	0.667	2.167	1.000	1.833	A
	1.000	2.000	1.000	2.000	0.667	2.167	1.000	2.000	G

### 2.2. Comparative dN/dS calculation for a sample of 29 genes in *E. coli* and *Salmonella enterica*

We then tested this method on a sample set of 29 genes in 100 genomes of two closely related Gram-negative bacteria i.e. *E. coli* and *Salmonellaenterica*. These genes are of same size in both the bacteria having no insertion or deletion mutations (Supplementary Tables S2 and S5). Comparisons were made among the dN/dS values in the sample 29 genes using their reference sequences by three methods, (i) Nei–Gojobori method, (ii) the MEGA-X, and (iii) The proposed method in this study.

### 2.3. Software and programme

Programme written in Python-language is available at GitHub in the following link. (https://github.com/Debapriya-Tula/calculating_dN-dS, 8 July 2022, date last accessed). Further queries regarding computer programme may contact SSS (ssankar@tezu.ernet.in).

## 3. Results

Synonymous substitution frequency in TFD codons found to be higher than FFD codons which is also termed as observed substitutions in a gene sequence. Now, to calculate the expected values synonymous and non-synonymous polymorphisms for individual codons, we obtained the S_o_ and NS_o_ changes in the 586 genes across the 156 strains of *E. coli*. As expected, S_o_ was higher than the NS_o_ in case of every codon. This is expected considering the purifying selection being stronger on NS than on S (Supplementary Table S1). We calculated the expected number of S (S_e_) and expected number of NS (NS_e_) for each codon from their S_o_ and NS_o_ values (Materials and Methods). The S_o_/S_e_, which was defined as fS, was found out for each codon to normalize the value so that we could do a comparative study across the 59 codons regarding synonymous changes (Supplementary Table S1).

**Figure 1:**
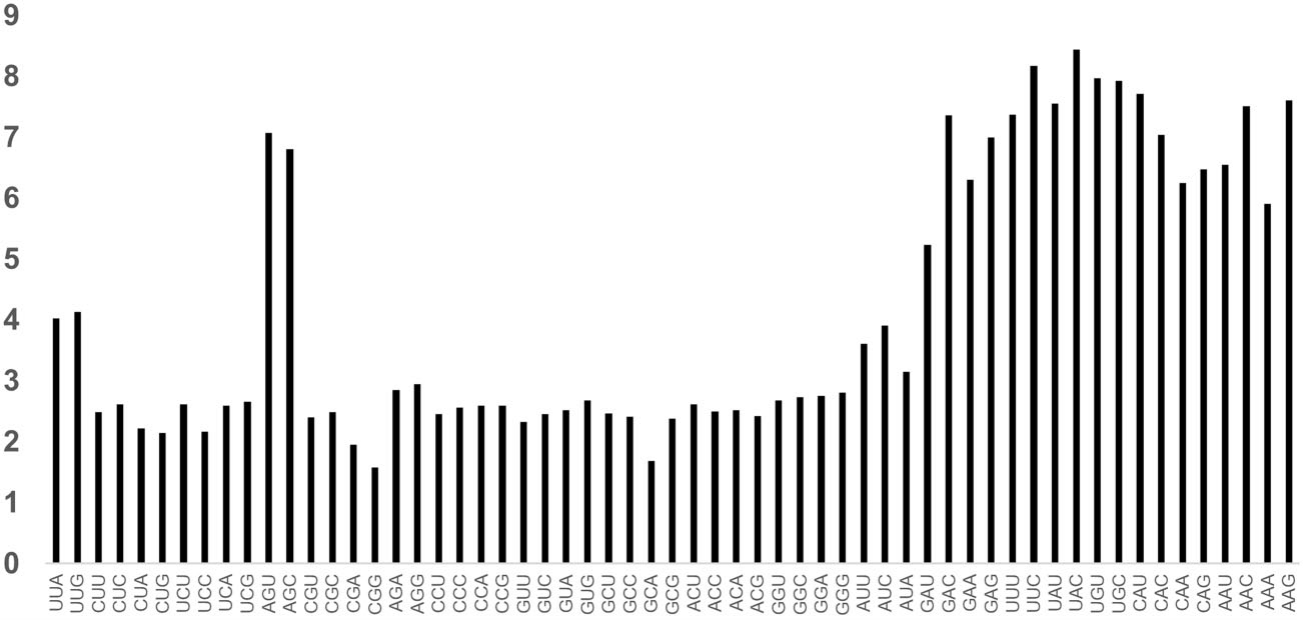
fS values of different codons in *E. coli*. Ratio between the observed number of synonymous polymorphisms to the expected number of synonymous polymorphisms (fS values) observed in all codons to that of expected number in the codon. The 59 codons are on the *x*-axis. The vertical bar represents the fS values of individual codons. It is evident that the values of TFD codons (in the right side of the graph) are higher than that of the FFD codons. In case of Ser, fS values of the split box codons (AGY) are like that of TFD codons, whereas the same of the family box codons (UCN) are similar that of FFD codons.

**Figure 2:**
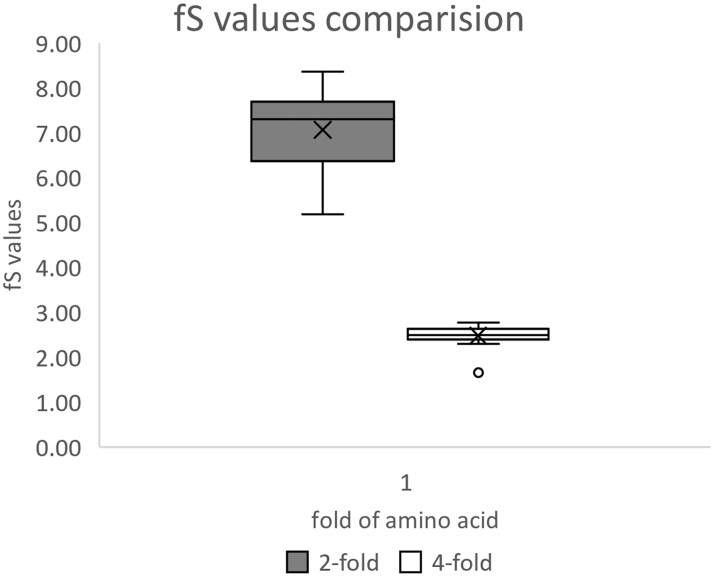
Box-plot comparison of fS values between TFD and FFD. Box-plot between fS values (So/Se) of synonymous polymorphism observed across TFD and FFD codons. The fS value of 2FD codons ranges between 5.19 and 8.36, with mean 7.0713, s.d. 7.3, whereas that of FFD codons ranges between 2.3 and 2.77, with mean 2.48, s.d. 2.49. The values are significantly different (*P* < 0.01) between TFD and FFD. https://www.socscistatistics.com/tests/mannwhitney/default2.aspx (25 June 2022, date last accessed) site was used for the Mann–Whitney *U*-test.

The calculated fS values were variable across the 59 codons in the *E. coli* genome (Fig. 1). Codon degeneracy has an impact on fS values, with distinct low values for FFD codons and high values for TFD codons. TFD amino acid codons Phe, Tyr, Cys, His, Gln, Asn, Lys, Asp, and Glu exhibited 2-fold higher fS values than FFD amino acid codons Val, Pro, Thr, Ala, and Gly (Figs 1 and 2; *P*-value < 0.01). We found variable observations for amino acids like Leu, Arg, and Ser (6-fold degenerate codons). In case of Ser, the fS values of family box codons exhibited like FFDs, while the split box codon values behave like the TFDs. Critical analysis suggested that high fS values observed in TFD codons could be attributed due to lack of synonymous transversion (Fig. 2). Analysis of fS suggested that Ti/Tv values are influenced by codon degeneracy. This stimulated us to calculate the S and NS site values considering Ti/Tv substitution ratio in different codons.

In this study, modified calculation of S and NS sites of individual codons accounts transition and transversion rate difference and nonsense substitutions in pretermination codon. The transition and transversion rates are implemented considering the assumed variable for all S and NS site in codons (Table 2). For example, at the S site in the genetic code table considers both CUG and AGG codons are similar with the value 1.333. However, in synonymous polymorphism in CUG is more than AGG because there are more synonymous substitutions due to transition in codon CUG (Supplementary Table S4). A nonsense mutation in a codon apparently makes all the downstream codons non-functional. This also suggests that it is probably the codons corresponding to pretermination codons might influence substitutions in a gene. Therefore, substitution frequency in pretermination codons is likely to be observed less than the other codons. This might be an explanation for the observation of lower substitution in UGG (Trp) codon in comparison to the AUG (Met) codon.^22^^,^^23^ Similar explanations can be given for other pretermination codons. In case of UUA and UUG, the NS sites should be considered differently despite both being pretermination codons because in case of UUA two different substitutions results nonsense codons while in case UUG only one substitution results in a nonsense codon. Therefore, we implemented the rate difference between Ti and Tv and accounted the nonsense mutations in pretermination codons in calculation of S and NS values in codons.

The calculated S and NS site values of codons using the modified method differs due to consideration of Ti and Tv rate difference and non-synonymous substitutions occurring due to pretermination codons (Supplementary Table S4). The calculated values given in modified Tables 1 and 2 provide easy reference for understanding the rate of synonymous substitutions in different codons.^24^^,^^25^ It is pertinent to note that this approach is not limited to organisms *E. coli* and *S. enterica* whose Ti is four times more than Tv but is nearly applicable for organisms with alternative Ti/Tv ratio. Regarding non-synonymous substitutions, the obvious difference is observed between AUG (Met) and UGG (Trp) which is a pretermination codon. In case of UGG, two (transitions) out of nine substitutions result in nonsense codon. In Table 1, the NS site values are considered as 3.0 for AUG and UGG, suggesting that both the codons should have equal frequencies of NS substitutions. However, modified the NS site value for AUG is considered as 3.0, while for UGG is considered as 1.667 as calculated using the modified method (Table 2). To have a further insight into the application of our method we compared non-synonymous substitutions in AUG and UGG codons. Total number of AUG codon in the 586 genes were 4,255 excluding the initiation codon, whereas the number of UGG codon were 2,441. In case of AUG, number of NS changes was observed as 914, whereas the same in case of UGG was 61. Considering the NS values equal for both the codons, it can be calculated that the frequency of non-synonymous changes in AUG is 8.5 times higher than that of UGG. But in the modified approach here the NS site for AUG and UGG are 3.0 and 1.667, respectively. Therefore, the frequency of non-synonymous changes in AUG is only 4.5 times than that of UGG. Hence, the accuracy of data can be improved by considering the Ti/Tv ratio and nonsense substitutions.

As per the modified method proposed in this study, the total synonymous site value for all the codons in the genetic code table is 53.333 (∼30%), while the total synonymous site value for all the codons in the genetic code table by the earlier method is 44.664 (∼25%) indicating an increase in the synonymous site values by the modified method might be due to higher proportion of synonymous changes attributed to transitions (Tables 1 and 2). Further, sum of all the non-synonymous sites value is 123.335 (∼70%) in the modified calculation while the same is 138.336 (∼75%) in the earlier method, indicating the decrease in the non-synonymous sites is due to consideration of nonsense sites in the pretermination codons in the modified method. Therefore, the dN/dS values of gene sequences estimated by modified method are higher than the existing method. A computer programme is developed considering the proposed modification in calculating the S and NS sites.

### 3.1. Comparative dN/dS values calculated using the new S and NS sites codons is influenced by codon degeneracy and pretermination codons

**Figure 3:**
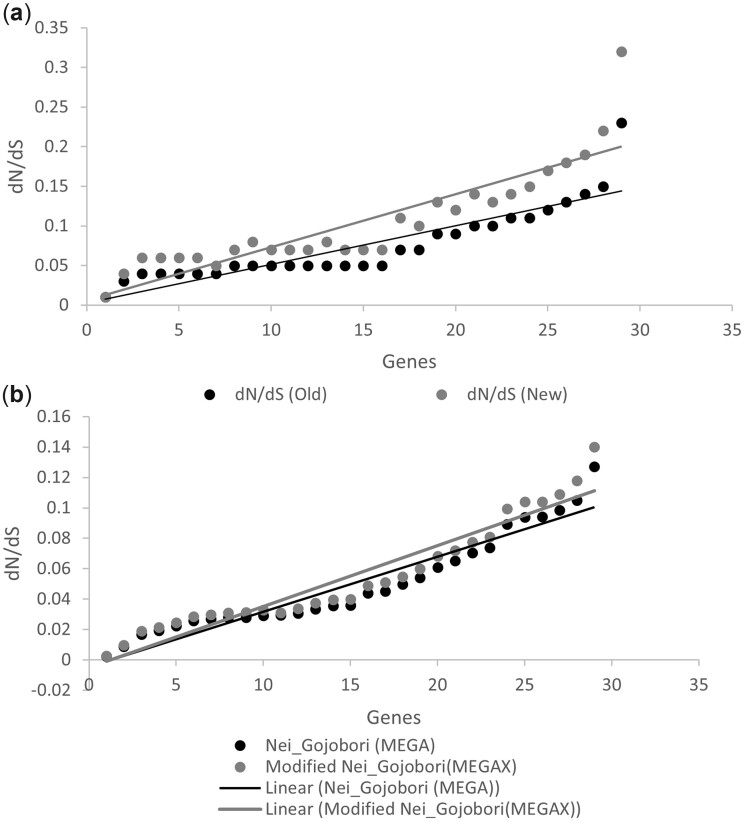
(a) scattered plot of the dN/dS values of the sample 29 genes by the proposed method. (b) Scattered plot of the dN/dS values of the sample 29 genes using MEGA-X. In (a), dN/dS values of the sample 29 genes between *E. coli* and *S. enterica* were calculated using the simple approach vs the proposed method in this study (Materials and Methods). dN/dS in the proposed method accounting Ti/Tv and nonsense substitutions is higher than that of the old method in every gene. We have only considered single substitutions in the calculation of dN/dS. In (b), Scattered-plot of dN/dS values of the sample 29 genes using MEGA-X (Nei-Gojobori dN/dS method and modified-Nei-Gojobori dN/dS method (considering Ti/Tv ratio = 2) in the same 29 genes of *E. coli* and *S. enterica* in MEGA-X, although in these methods double and triple mutations are also considered along with the single substitutions.

We then tested modified method in a sample set of 29 common genes in 100 genomes each from two closely related Gram-negative bacteria i.e. *E. coli* and *S. enterica*. These genes are of same size in both the bacteria having no insertion or deletion mutations. We calculated dN/dS by the S and NS sites using existing and modified method. It is evident from the result that the dN/dS values using the new calculation is higher than that calculated by the previous method in each of the 29 genes (Fig. 3a andSupplementary Table S2). We then calculated the dN/dS values of 29 genes common to *E. coli* and *S. enterica* using our modified method and demonstrated that pretermination codons play an important role in estimating the accuracy of dN/dS values.

Further, we found out percentage increase in the dN/dS values in each gene by established method, but with altered values. Among these genes, the increase in dN/dS values range from 29% to 50%. To explore the reason for this difference, by finding out the number of TFD codons vs number of FFD codons. If the proportion of TFD is more than the FFD, then the difference values are likely to be more. We found out the ratio of TFD and FFD in each of the 29 genes (Supplementary Table S2). The minimum ratio is 0.680:1.000, while the maximum ratio was 1.718:1.000. We did a correlation between the difference values with the TFD:FFD ratio. The Pearson *r* was observed as 0.931 of the correlation between difference values vs the ratio of TFD:FFD codons in each gene. This proved our approach is considering the degeneracy of the composition of codons varies from 16.0% to 36.0% across these 29 genes. We also found out the correlation between pretermination codon composition (%) in a gene and the percentage increase in the dN/dS values calculated by the new method. The Pearson *r* value was 0.651 suggesting the impact of pretermination codon composition in a gene on the dN/dS value. This proved the new values have combined influenced on dN/dS due to the composition of pretermination codon and Ti/Tv substitution rate. It is pertinent to note that recently Nei–Gojobori method has been modified to incorporate Ti and Tv (MEGA-X). Using Nei and Gojobori (MEGA-X) and Nei and Gojobori modified (MEGA-X), dN/dS values of the 29 genes were calculated and compared (Fig. 3b and Supplementary Table S3).^26–28^ The dN/dS values by Nei and Gojobori modified were higher than that calculated by Nei and Gojobori, as expected. The correlation value between the % increase and the ratio between TFD: FFD codons was 0.726 (Pearson *r* value), which was lesser than Pearson *r* value 0.93 found out above using the modified method. Further, the correlation value between the percentage increase and the fraction of pretermination codons in genes was 0.51 (Pearson *r* value), which was less than Pearson *r* value 0.64, found out above using the modified method. This indicates our proposed method might account more accurately the Ti/Tv rates as well as pretermination codons while estimating the dN/dS values.

## 4. Discussion

In this study, we have demonstrated that synonymous dynamics in TFD codons are comparatively higher than that in FFD codons considering *E. coli* genome as a model. Further, we have demonstrated that within 6-fold degenerate codons, synonymous dynamics in split box codons are comparatively higher than that from the family box codons. The difference in synonymous dynamics regarding codon degeneracy can be attributed to the higher rate of transition than transversion substitution mutation in organisms. In this study, the transition and transversion rate differences have been implemented in calculating S and NS sites in codons in the genetic code table. We also have included the significance of nonsense substitutions while calculating the NS sites in case of the eighteen pre-terminations codons. Although, low frequency of non-synonymous substitutions in Trp and Cys codons have been attributed of the possible positional significance of these amino acids in protein function,^29^ the low NS sites due to pretermination nature of these codons determined using the modified method described in the present study can be an additional explanation.

By incorporating Ti/Tv substitution rate difference in codons as well as nonsense substitutions in pretermination codons, the dN/dS values estimated in a sample of 29 genes suggested the role of codon degeneracy and pretermination codon compositions. Though dN/dS is a widely used approach to study selection on genes, various models were proposed till date with substantial evidence to justify the role of substitution on codon sites across genes.^30^ But still, it is a matter of intrigue and examination because of the occurrence of certain constant or hyper-transient sites in the sequences highlighting the importance of analysis of individual codons. Recently, it has been demonstrated that the weak selection on synonymous codons substantially inflates dN/dS rates in bacteria.^31^ In this study, the modified method of calculating dN/dS provides a simple biological understanding of variability of genes to comprehend codon assignment of the codons in the genetic code table. In this study role of pretermination codon is included and impact of nonsense mutations justifies not only the rate of nonsynonymous/synonymous substitution ratio in the genome but also the effect of the codon usage biasness dominating the equilibrium of codon composition. This helps in predicting and comparing the gene variations of a genome. Consideration of individual substitution rates in codons is expected to further complicate the estimation but should improve accuracy of the dN/dS.

In conclusion, here we have elucidated that by accounting Ti/Tv substitution rates and nonsense substitutions in pretermination codon in calculating S and NS site in codons have resulted in increase of the dN/dS values in gene sequences. Considering the simplicity and biological relevance of the proposed method to estimate dN/dS in genes might contribute wider scope in evolutionary studies. This work sheds light on the robustness of the composition of amino acids with different codon degeneracy as well as pretermination nature of codons in calculation of the dN/dS values. Further in this study, we have estimated the dN/dS between the two closely related species *E. coli* and *S. enterica* where Ti/Tv ratio in these two organisms are similar. It will be an interesting study in future considering distantly related bacteria having different Ti/Tv ratios as the modified method emphasizes non-synonymous site calculation due to pretermination codons.

## Supplementary data

Supplementary data are available at DNARES online.

## Authors contributions

R.A.: executed, data analysis, writing, and discussion; P.S.: executed, data analysis, and writing; P.K.B.: executed, writing, and discussion; S.D.: discussion; D.T. and M.D.: computer programming and execution; N.D.N., R.C.D., and EF: critical analysis, writing, and discussion; SSS, SKR: design, critical analysis, writing, and discussion.

## Supplementary Material

dsac023_Supplementary_Data
