# Supplementary material for: Incorporation of transition to transversion ratio and nonsense mutations, improves the estimation of the number of synonymous and non-synonymous sites in codons
Source: DNA Res. 2022 Aug 3;29(4):dsac023. doi: 10.1093/dnares/dsac023 (PMC9358017; doi:10.1093/dnares/dsac023)
Supplement: dsac023_Supplementary_Data [file dsac023_supplementary_data.docx]

**Supplementary Table 1: Synonymous polymorphisms (fS) in 156 strains *E. coli* 586 genes from the whole genome**

|  | **Codons** | **S_e_ (Expected)** | **NS_e_ (Expected)** | **S_o_ (Observed)** | **NS_o_ (Observed)** | **Total (S_o_+NS_o_)** | **fS (S_o_/S_e_)** |
| --- | --- | --- | --- | --- | --- | --- | --- |
| **L** | UUA | 88.71 | 310.29 | 353.00 | 46.00 | 399.00 | 3.98 |
|  | UUG | 168.75 | 590.25 | 690.00 | 69.00 | 759.00 | 4.09 |
|  | CUU | 132.33 | 264.67 | 325.00 | 72.00 | 397.00 | 2.46 |
|  | CUC | 170.33 | 340.67 | 440.00 | 71.00 | 511.00 | 2.58 |
|  | CUA | 138.19 | 172.78 | 303.00 | 8.00 | 311.00 | 2.19 |
|  | CUG | 1305.01 | 1631.67 | 2766.00 | 171.00 | 2937.00 | 2.12 |
| **S** | UCU | 119.33 | 238.67 | 309.00 | 49.00 | 358.00 | 2.59 |
|  | UCC | 63.33 | 126.67 | 136.00 | 54.00 | 190.00 | 2.15 |
|  | UCA | 97.33 | 194.67 | 250.00 | 42.00 | 292.00 | 2.57 |
|  | UCG | 162.33 | 324.67 | 426.00 | 61.00 | 487.00 | 2.62 |
|  | AGU | 33.52 | 268.44 | 235.00 | 67.00 | 302.00 | 7.01 |
|  | AGC | 83.36 | 667.56 | 562.00 | 189.00 | 751.00 | 6.74 |
| **R** | CGU | 314.33 | 628.67 | 748.00 | 195.00 | 943.00 | 2.38 |
|  | CGC | 449.00 | 898.00 | 1105.00 | 242.00 | 1347.00 | 2.46 |
|  | CGA | 87.09 | 108.91 | 168.00 | 28.00 | 196.00 | 1.93 |
|  | CGG | 81.76 | 102.24 | 128.00 | 56.00 | 184.00 | 1.57 |
|  | AGA | 13.10 | 45.90 | 37.00 | 22.00 | 59.00 | 2.82 |
|  | AGG | 8.21 | 28.79 | 24.00 | 13.00 | 37.00 | 2.92 |
| **P** | CCU | 120.67 | 241.33 | 293.00 | 69.00 | 362.00 | 2.43 |
|  | CCC | 99.33 | 198.67 | 252.00 | 46.00 | 298.00 | 2.54 |
|  | CCA | 185.00 | 370.00 | 474.00 | 81.00 | 555.00 | 2.56 |
|  | CCG | 467.33 | 934.67 | 1200.00 | 202.00 | 1402.00 | 2.57 |
| **V** | GUU | 251.33 | 502.67 | 579.00 | 175.00 | 754.00 | 2.30 |
|  | GUC | 313.67 | 627.33 | 762.00 | 179.00 | 941.00 | 2.43 |
|  | GUA | 256.00 | 512.00 | 639.00 | 129.00 | 768.00 | 2.50 |
|  | GUG | 458.00 | 916.00 | 1214.00 | 160.00 | 1374.00 | 2.65 |
| **A** | GCU | 306.67 | 613.33 | 747.00 | 173.00 | 920.00 | 2.44 |
|  | GCC | 551.67 | 1103.33 | 1317.00 | 338.00 | 1655.00 | 2.39 |
|  | GCA | 203.67 | 407.33 | 339.00 | 272.00 | 611.00 | 1.66 |
|  | GCG | 806.00 | 1612.00 | 1896.00 | 522.00 | 2418.00 | 2.35 |
| **T** | ACU | 198.67 | 397.33 | 513.00 | 83.00 | 596.00 | 2.58 |
|  | ACC | 464.33 | 928.67 | 1149.00 | 244.00 | 1393.00 | 2.47 |
|  | ACA | 122.00 | 244.00 | 304.00 | 62.00 | 366.00 | 2.49 |
|  | ACG | 302.33 | 604.67 | 724.00 | 183.00 | 907.00 | 2.39 |
| **G** | GGU | 451.67 | 903.33 | 1196.00 | 159.00 | 1355.00 | 2.65 |
|  | GGC | 658.33 | 1316.67 | 1780.00 | 195.00 | 1975.00 | 2.70 |
|  | GGA | 136.33 | 272.67 | 372.00 | 37.00 | 409.00 | 2.73 |
|  | GGG | 220.00 | 440.00 | 610.00 | 50.00 | 660.00 | 2.77 |
| **I** | AUU | 157.41 | 550.67 | 562.00 | 146.00 | 708.00 | 3.57 |
|  | AUC | 231.45 | 809.67 | 895.00 | 146.00 | 1041.00 | 3.87 |
|  | AUA | 28.90 | 101.11 | 90.00 | 40.00 | 130.00 | 3.11 |
| **D** | GAU | 85.80 | 687.11 | 445.00 | 328.00 | 773.00 | 5.19 |
|  | GAC | 109.22 | 874.67 | 797.00 | 187.00 | 984.00 | 7.30 |
| **E** | GAA | 99.35 | 795.56 | 620.00 | 275.00 | 895.00 | 6.24 |
|  | GAG | 84.69 | 678.22 | 587.00 | 176.00 | 763.00 | 6.93 |
| **F** | UUU | 35.19 | 281.78 | 257.00 | 60.00 | 317.00 | 7.30 |
|  | UUC | 55.83 | 447.11 | 452.00 | 51.00 | 503.00 | 8.10 |
| **Y** | UAU | 32.86 | 263.11 | 246.00 | 50.00 | 296.00 | 7.49 |
|  | UAC | 51.06 | 408.89 | 427.00 | 33.00 | 460.00 | 8.36 |
| **C** | UGU | 20.76 | 166.22 | 164.00 | 23.00 | 187.00 | 7.90 |
|  | UGC | 35.63 | 285.33 | 280.00 | 41.00 | 321.00 | 7.86 |
| **H** | CAU | 97.90 | 784.00 | 748.00 | 134.00 | 882.00 | 7.64 |
|  | CAC | 45.73 | 366.22 | 319.00 | 93.00 | 412.00 | 6.98 |
| **Q** | CAA | 44.29 | 354.67 | 274.00 | 125.00 | 399.00 | 6.19 |
|  | CAG | 104.56 | 837.33 | 671.00 | 271.00 | 942.00 | 6.42 |
| **N** | AAU | 48.73 | 390.22 | 316.00 | 123.00 | 439.00 | 6.48 |
|  | AAC | 90.47 | 724.44 | 673.00 | 142.00 | 815.00 | 7.44 |
| **K** | AAA | 50.73 | 406.22 | 297.00 | 160.00 | 457.00 | 5.85 |
|  | AAG | 38.63 | 309.33 | 291.00 | 57.00 | 348.00 | 7.53 |
| **M** | AUG | 0.00 | 914.00 | 0.00 | 914.00 | 914.00 | 0.00 |
| **W** | UGG | 0.00 | 61.00 | 0.00 | 61.00 | 61.00 | 0.00 |

S_e_: number of synonymous changes expected for a codon

NS_e_: number of non-synonymous changes expected for a codon

S_o_: number of synonymous changes observed for a codon

NS_o_: number of non-synonymous changes observed for a codon

The observed values are synonymous and non-synonymous changes found out by comparing 586 gene sequences across 156 *E. coli* strains. The expected values are calculated by multiplying the total number of changes with the fraction of single substitution in a codon that results either synonymous or non-synonymous.

**Supplementary Table 2: A comparison of dN/dS values by the old and the new methods in 29 genes of *Escherichia coli* and *Salmonella enterica*** Ratio (TFD:FFD): Two fold degenerate site (TFD); Four-fold degenerate site (FFD). Freq PreTer: Fraction of pre-termination codons in a gene. List of eighteen pretermination codons: UUA, UUG, UCA, UCG, UAU, UAC, UGU, UGC, UGG, CAA, CAG, AAA, AAG, GAA, GAG, CGA, AGA, GGA

| **Sl** | **Gene** | **Number of S Mutation** | **Number of NS Mutation** | **S site (New)** | **NS site (New)** | **dN/dS (New)** | **S site (Old)** | **NS site (Old)** | **dN/dS (Old)** | **Diff** | **% increase** | **Ratio** | **Freq PreTer codons** |
| --- | --- | --- | --- | --- | --- | --- | --- | --- | --- | --- | --- | --- | --- |
| 1 | *araE* | 223 | 23 | 410.42 | 966.84 | 0.04 | 340.83 | 1078.17 | 0.03 | 0.011 | 34.280 | 0.680 | 0.205 |
| 2 | *gltI* | 133 | 20 | 256.25 | 621.43 | 0.06 | 202.50 | 706.50 | 0.04 | 0.019 | 43.867 | 1.337 | 0.274 |
| 3 | *gltR_1* | 171 | 76 | 252.75 | 591.01 | 0.19 | 209.50 | 668.00 | 0.14 | 0.051 | 36.361 | 0.846 | 0.248 |
| 4 | *gltX* | 171 | 30 | 398.58 | 971.35 | 0.07 | 314.67 | 1101.33 | 0.05 | 0.022 | 43.619 | 1.366 | 0.248 |
| 5 | *hemA* | 163 | 28 | 370.92 | 840.93 | 0.08 | 299.83 | 957.17 | 0.05 | 0.022 | 40.807 | 1.111 | 0.265 |
| 6 | *hemB* | 149 | 27 | 277.58 | 671.01 | 0.07 | 226.17 | 748.83 | 0.05 | 0.020 | 36.969 | 1.037 | 0.255 |
| 7 | *hemC* | 145 | 45 | 291.33 | 645.43 | 0.14 | 244.17 | 718.83 | 0.11 | 0.035 | 32.888 | 0.774 | 0.224 |
| 8 | *hemD* | 82 | 57 | 224.00 | 480.42 | 0.32 | 182.00 | 559.00 | 0.23 | 0.098 | 43.207 | 1.068 | 0.328 |
| 9 | *hemG* | 79 | 30 | 150.33 | 370.67 | 0.15 | 121.67 | 424.33 | 0.11 | 0.045 | 41.448 | 1.302 | 0.302 |
| 10 | *hemH* | 144 | 46 | 283.50 | 644.84 | 0.14 | 234.00 | 729.00 | 0.10 | 0.038 | 36.965 | 0.927 | 0.265 |
| 11 | *hemN_1* | 175 | 51 | 330.42 | 762.26 | 0.13 | 262.33 | 874.67 | 0.09 | 0.039 | 44.527 | 1.246 | 0.280 |
| 12 | *hemN_2* | 185 | 46 | 394.83 | 925.68 | 0.11 | 304.67 | 1069.33 | 0.07 | 0.035 | 49.707 | 1.718 | 0.288 |
| 13 | *hisB* | 149 | 23 | 302.67 | 732.93 | 0.06 | 238.33 | 829.67 | 0.04 | 0.019 | 43.754 | 1.408 | 0.247 |
| 14 | *hisF* | 102 | 18 | 220.08 | 530.68 | 0.07 | 180.67 | 596.33 | 0.05 | 0.020 | 36.888 | 1.044 | 0.232 |
| 15 | *lacG* | 109 | 30 | 242.92 | 578.00 | 0.12 | 202.83 | 643.17 | 0.09 | 0.029 | 33.263 | 0.680 | 0.163 |
| 16 | *leuE* | 119 | 45 | 192.42 | 427.34 | 0.17 | 158.33 | 480.67 | 0.12 | 0.046 | 36.692 | 0.868 | 0.249 |
| 17 | *leuS* | 278 | 50 | 720.42 | 1772.37 | 0.07 | 582.83 | 2000.16 | 0.05 | 0.021 | 39.491 | 1.112 | 0.259 |
| 18 | *malF* | 196 | 60 | 453.33 | 1037.51 | 0.13 | 366.67 | 1178.33 | 0.10 | 0.039 | 40.418 | 0.917 | 0.237 |
| 19 | *pepN* | 354 | 66 | 749.75 | 1779.12 | 0.08 | 595.00 | 2017.99 | 0.05 | 0.024 | 42.925 | 1.303 | 0.240 |
| 20 | *polA* | 440 | 74 | 817.50 | 1885.80 | 0.07 | 658.50 | 2128.50 | 0.05 | 0.021 | 40.124 | 1.086 | 0.279 |
| 21 | *recO* | 109 | 23 | 219.42 | 486.51 | 0.10 | 178.83 | 550.17 | 0.07 | 0.027 | 38.747 | 0.975 | 0.247 |
| 22 | *recR* | 73 | 12 | 177.33 | 408.01 | 0.07 | 146.67 | 459.33 | 0.05 | 0.019 | 36.121 | 0.971 | 0.257 |
| 23 | *rpoC* | 216 | 27 | 1265.75 | 2858.56 | 0.06 | 1041.00 | 3183.01 | 0.04 | 0.014 | 35.391 | 0.996 | 0.223 |
| 24 | *rpoE* | 37 | 1 | 166.33 | 386.42 | 0.01 | 134.17 | 441.83 | 0.01 | 0.003 | 41.757 | 1.175 | 0.302 |
| 25 | *rpoH* | 79 | 11 | 242.08 | 578.93 | 0.06 | 192.17 | 662.83 | 0.04 | 0.018 | 44.233 | 1.397 | 0.260 |
| 26 | *rpoN* | 186 | 31 | 413.08 | 963.35 | 0.07 | 324.67 | 1109.33 | 0.05 | 0.023 | 46.513 | 1.411 | 0.291 |
| 27 | *topA_2* | 68 | 36 | 153.50 | 366.92 | 0.22 | 122.00 | 421.00 | 0.15 | 0.068 | 44.362 | 1.379 | 0.365 |
| 28 | *trpA* | 138 | 56 | 241.83 | 541.93 | 0.18 | 199.67 | 607.33 | 0.13 | 0.048 | 35.736 | 0.865 | 0.249 |
| 29 | *trpS* | 108 | 14 | 288.83 | 681.35 | 0.05 | 227.17 | 777.83 | 0.04 | 0.017 | 45.153 | 1.308 | 0.272 |
| Min |  | 37 | 1 | 150.33 | 366.92 | 0.01 | 121.67 | 421.00 | 0.01 | 0.003 | 32.888 | 0.680 | 0.163 |
| Max |  | 440 | 76 | 1265.75 | 2858.56 | 0.32 | 1041.00 | 3183.01 | 0.23 | 0.098 | 49.707 | 1.718 | 0.365 |

**Supplementary Table 3: dN/dS values calculated by MEGA and MEGAX in a sample of 29 genes of *E. coli* and *S. enterica***

| **Sl No** | **Genes** | **Nei_Gojobori (MEGA-X) dN/dS** | **Modified Nei_Gojobori dN/dS** | **difference** | **% increase** | **Ratio (TFD:FFD)** | **Fraction of PreTer codon** |
| --- | --- | --- | --- | --- | --- | --- | --- |
| 1 | araE | 0.0224 | 0.0239 | 0.0015 | 6.696 | 0.68 | 0.205 |
| 2 | gltI | 0.0985 | 0.106 | 0.0075 | 7.614 | 1.337 | 0.274 |
| 3 | gltR_1 | 0.127 | 0.136 | 0.009 | 7.087 | 0.846 | 0.248 |
| 4 | gltX | 0.0279 | 0.0301 | 0.0022 | 7.885 | 1.366 | 0.248 |
| 5 | hemA | 0.0296 | 0.032 | 0.0024 | 8.108 | 1.111 | 0.265 |
| 6 | hemB | 0.0361 | 0.0387 | 0.0026 | 7.202 | 1.037 | 0.255 |
| 7 | hemC | 0.0653 | 0.0699 | 0.0046 | 7.044 | 0.774 | 0.224 |
| 8 | hemD | 0.105 | 0.114 | 0.009 | 8.571 | 1.068 | 0.328 |
| 9 | hemG | 0.0738 | 0.0788 | 0.005 | 6.775 | 1.302 | 0.302 |
| 10 | hemH | 0.0704 | 0.0753 | 0.0049 | 6.960 | 0.927 | 0.265 |
| 11 | hemN_1 | 0.061 | 0.0661 | 0.0051 | 8.361 | 1.246 | 0.28 |
| 12 | hemN_2 | 0.0451 | 0.0491 | 0.004 | 8.869 | 1.718 | 0.288 |
| 13 | hisB | 0.0279 | 0.0302 | 0.0023 | 8.244 | 1.408 | 0.247 |
| 14 | hisF | 0.0307 | 0.0328 | 0.0021 | 6.840 | 1.044 | 0.232 |
| 15 | lacG | 0.0498 | 0.0532 | 0.0034 | 6.827 | 0.68 | 0.163 |
| 16 | leuE | 0.0943 | 0.101 | 0.0067 | 7.105 | 0.868 | 0.249 |
| 17 | leuS | 0.0259 | 0.0277 | 0.0018 | 6.950 | 1.112 | 0.259 |
| 18 | malF | 0.0542 | 0.0583 | 0.0041 | 7.565 | 0.917 | 0.237 |
| 19 | pepN | 0.0336 | 0.0363 | 0.0027 | 8.036 | 1.303 | 0.24 |
| 20 | polA | 0.0356 | 0.0384 | 0.0028 | 7.865 | 1.086 | 0.279 |
| 21 | recO | 0.0439 | 0.0473 | 0.0034 | 7.745 | 0.975 | 0.247 |
| 22 | recR | 0.027 | 0.029 | 0.002 | 7.407 | 0.971 | 0.257 |
| 23 | rpoC | 0.00872 | 0.00935 | 0.00063 | 7.225 | 0.996 | 0.223 |
| 24 | rpoE | 0.00232 | 0.00249 | 0.00017 | 7.328 | 1.175 | 0.302 |
| 25 | rpoH | 0.017 | 0.0184 | 0.0014 | 8.235 | 1.397 | 0.26 |
| 26 | rpoN | 0.0293 | 0.0318 | 0.0025 | 8.532 | 1.411 | 0.291 |
| 27 | topA_2 | 0.0892 | 0.0964 | 0.0072 | 8.072 | 1.379 | 0.365 |
| 28 | trpA | 0.094 | 0.101 | 0.007 | 7.447 | 0.865 | 0.249 |
| 29 | trpS | 0.0192 | 0.0208 | 0.0016 | 8.333 | 1.308 | 0.272 |

**SupplementaryTable4: Examples to calculate S and NS for the modified method**

| **Codon** | **Three possible substitutions at the 1st codon position** | | | | |
| --- | --- | --- | --- | --- | --- |
| **UUU(F)** | CUU(L) | AUU(I) | GUU(V) | (Avg S site score) | (Avg NS site score) |
| Two-fold degenerate codon | NSTi | NSTv | NSTv |  |  |
|  | 4 | 1 | 1 | 0 | 1 |
|  | **Three possible substitutions at the 2nd codon position** | | | | |
|  | UCU(S) | UAU(Y) | UGU (C) |  |  |
|  | NSTi | NSTv | NSTv |  |  |
|  | 4 | 1 | 1 | 0 | 1 |
|  | **Three possible substitutions at the 3rd codon position** | | | | |
|  | UUC(F) | UUA(L) | UUG (L) |  |  |
|  | Sti | NSTv | NSTv |  |  |
|  | 4 | 1 | 1 | 0.667 | 0.333 |
| **total** |  | | | **0.667** | **2.333** |
|  |  |  |  |  |  |
|  | **Three possible substitutions at the 1st codon position** | | | | |
| **UUA(L)** | CUA(L) | AUA(I) | GUA(V) | (Avg S site score) | (Avg NS site score) |
| (pre-termination codon) | STi | NSTv | NSTv |  |  |
|  | 4 | 1 | 1 | 0.667 | 0.333 |
|  | **Three possible substitutions at the 2nd codon position** | | | | |
|  | UCA(Ser) | UAA(Stop) | UGA(Stop) |  |  |
|  | NSTi | NSTv | NSTv |  |  |
|  | 4 | 1 | 1 | 0 | 0.667 |
|  | **Three possible substitutions at the 3rd codon position** | | | | |
|  | UUU(F) | UUC(F) | UUG(L) |  |  |
|  | NStv | NSTv | Sti |  |  |
|  | 1 | 1 | 4 | 0.667 | 0.333 |
| **total** |  | | | **1.333** | **1.333** |
|  |  |  |  |  |  |
| **UUG(L)** | **Three possible substitutions at the 1st codon position** | | | | |
| (pre-termination codon) | CUG(L) | AUG(M) | GUG(V) | (Avg S site score) | (Avg NS site score) |
|  | STi | NSTv | NSTv |  |  |
|  | 4 | 1 | 1 | 0.667 | 0.333 |
|  | **Three possible substitutions at the 2nd codon position** | | | | |
|  | UCG(S) | UAG(Stop) | UGG(W) |  |  |
|  | NSTi | NSTv | NSTv |  |  |
|  | 4 | 1 | 1 | 0.8 | 0.2 |
|  | **Three possible substitutions at the 3rd codon position** | | | | |
|  | UUU(F) | UUC(F) | UUA(L) |  |  |
|  | NStv | NSTv | Sti |  |  |
|  | 1 | 1 | 4 | 0.667 | 0.333 |
| **total** |  | | | **2.133** | **0.867** |
|  |  |  |  |  |  |
| **GGU(G)** | **Three possible substitutions at the 1st codon position** | | | | |
| Four-fold degenerate codon | AGU(S) | CGU (R) | UGU(C) | (Avg S site score) | (Avg NS site score) |
|  | NSTi | NSTv | NSTv |  |  |
|  | 4 | 1 | 1 | 0 | 1 |
|  | **Three possible substitutions at the 2nd codon position** | | | | |
|  | GAU(D) | GCU (A) | GUU (V) |  |  |
|  | NSTi | NSTv | NSTv |  |  |
|  | 4 | 1 | 1 | 0 | 1 |
|  | **Three possible substitutions at the 3rd codon position** | | | | |
|  | GGC (G) | GGA (G) | GGG (G) |  |  |
|  | Sti | STv | Stv |  |  |
|  | 4 | 1 | 1 | 1 | 0 |
| **total** |  | | | **1** | **2** |

*ti* value is considered 4 and *tv* value as 1 as ti is four time more frequent tv. At every position out of three substitutions, one *ti* and two *tv*. ti value is 4 and tv value is 1. Total value is 4 +2 = 6. At every position, total Sti and Stv is divided by six that define S site at that position whereas total NSti and NStv is divided by 6 that define NS site at that position. In case of pretermination codons it is divided by 6 but the ti or tv mutations leading to non-sense codons are not counted.

**Supplementary Table 5: S and NS changes observed across 100 different strains of *E. coli* and *S. enterica***

| **Organisms** | | ***Escherichia coli*** | | | | | ***Salmonella enterica*** | | | | |
| --- | --- | --- | --- | --- | --- | --- | --- | --- | --- | --- | --- |
| **Genes** | **Amino Acids** | **(G+C)%** | **S obs** | **NS obs** | **Nc** | **CAI** | **(G+C)%** | **S obs** | **NS obs** | **Nc** | **CAI** |
| *araE* | 472 | 51.52 | 66 | 9 | 44.92 | 0.415 | 52.85 | 64 | 11 | 63.21 | 0.444 |
| *gltD_1* | 659 | 55.86 | 56 | 8 | 46.55 | 0.471 | 58.66 | 35 | 5 | 64.68 | 0.474 |
| *gltR_1* | 293 | 53.85 | 60 | 21 | 46.34 | 0.381 | 57.04 | 38 | 13 | 63.23 | 0.377 |
| *gltX* | 471 | 52.54 | 68 | 10 | 39.53 | 0.631 | 55.37 | 89 | 10 | 65.89 | 0.661 |
| *hemA* | 418 | 54.34 | 122 | 19 | 42.06 | 0.473 | 57.12 | 60 | 3 | 65.16 | 0.476 |
| *hemB* | 324 | 54.46 | 61 | 3 | 43.63 | 0.515 | 55.49 | 51 | 12 | 62.77 | 0.526 |
| *hemC* | 320 | 55.97 | 63 | 10 | 45.72 | 0.424 | 58.67 | 48 | 7 | 62.93 | 0.396 |
| *hemD* | 246 | 53.44 | 69 | 33 | 48.88 | 0.32 | 57.62 | 22 | 20 | 58.7 | 0.296 |
| *hemG* | 181 | 51.65 | 34 | 5 | 43.45 | 0.424 | 50.73 | 22 | 7 | 56.59 | 0.449 |
| *hemH* | 320 | 54.31 | 57 | 10 | 43.07 | 0.469 | 57.01 | 50 | 21 | 62.31 | 0.438 |
| *hemN_1* | 378 | 53.39 | 125 | 29 | 43.44 | 0.399 | 55.15 | 58 | 31 | 61.48 | 0.454 |
| *hemN_2* | 457 | 53.13 | 54 | 7 | 43.05 | 0.486 | 51.97 | 47 | 16 | 61.14 | 0.525 |
| *hisB* | 355 | 53.84 | 140 | 25 | 37.69 | 0.467 | 52.34 | 60 | 14 | 60.96 | 0.547 |
| *hisF* | 258 | 52.51 | 112 | 19 | 39.92 | 0.555 | 55.08 | 34 | 5 | 62.93 | 0.557 |
| *lacG* | 281 | 54.49 | 70 | 9 | 38.89 | 0.482 | 53.78 | 42 | 2 | 70.57 | 0.463 |
| *leuE* | 212 | 45.07 | 19 | 9 | 42.56 | 0.358 | 46.95 | 28 | 5 | 49.3 | 0.374 |
| *leuS* | 860 | 53.39 | 116 | 19 | 38.14 | 0.668 | 56.1 | 110 | 15 | 68.29 | 0.683 |
| *malF* | 514 | 53.33 | 75 | 13 | 39.47 | 0.474 | 52.69 | 78 | 13 | 65.83 | 0.476 |
| *pepN* | 870 | 54.08 | 192 | 20 | 40.78 | 0.493 | 53.73 | 106 | 29 | 62.23 | 0.558 |
| *polA* | 928 | 51.96 | 91 | 20 | 44.69 | 0.477 | 53.89 | 120 | 26 | 61.03 | 0.497 |
| *recO* | 242 | 53.91 | 44 | 5 | 42.34 | 0.408 | 55.28 | 36 | 8 | 61.73 | 0.415 |
| *recR* | 201 | 57.59 | 19 | 1 | 40.75 | 0.479 | 61.39 | 34 | 3 | 73.27 | 0.508 |
| *rpoC* | 1407 | 53.88 | 105 | 5 | 34.89 | 0.749 | 54.76 | 279 | 9 | 60.23 | 0.768 |
| *rpoE* | 191 | 48.78 | 6 | 1 | 46.72 | 0.392 | 51.04 | 10 | 1 | 54.69 | 0.357 |
| *rpoH* | 284 | 54.15 | 44 | 1 | 37.75 | 0.57 | 54.04 | 22 | 1 | 62.46 | 0.636 |
| *rpoN* | 477 | 53.14 | 59 | 9 | 46.08 | 0.432 | 54.04 | 56 | 6 | 63.18 | 0.462 |
| *topA_2* | 180 | 50.28 | 23 | 7 | 46.01 | 0.462 | 53.04 | 29 | 6 | 53.04 | 0.392 |
| *trpA* | 268 | 53.53 | 68 | 13 | 43.62 | 0.452 | 57.62 | 46 | 14 | 64.31 | 0.443 |
| *trpS* | 334 | 52.64 | 56 | 5 | 40 | 0.583 | 51.94 | 60 | 7 | 60.6 | 0.641 |

It is obvious to note that number S and NS changes among the strains within a species given in this Table in *E. coli* and in *S. enterica* is lower than the S and NS values across the two bacterial species given in Table2. CAI^21^ is calculated considering organism-specific ribosomal protein-coding genes as the reference set, in each bacterium using a web-based tool^22^. Nc calculation has been done as defined in the references^23^.
